# Mr. Edward Mason Wrench

**Published:** 1912-04-27

**Authors:** 


Mr. Edward Mason Wrench, M.V.O., F.R.C.S., consulting surgeon to the Whitworth Hospital, Darley, has died suddenly from heart failure while cycling. He was an assistant surgeon in the Crimea in 1854, and saw much active service at the front, while in 1856 he served in the Indian Mutiny as well. After leaving the army he settled in practice at Baslow; he soon became a prominent figure in the district and attended three Dukes of Devonshire at Chatsworth. Mr. Wrench took an active part in the volunteer movement, joining the 2nd Batt. Derbyshire Volunteers, of which he became Surg.-Lieut.-Colonel. He was also a J.P. for the county. His medical education was obtained at St. Thomas's Hospital, where he qualified in 1854.

